# Psychological Distress in the Galapagos Islands During the COVID-19 Pandemic

**DOI:** 10.3389/ijph.2022.1604366

**Published:** 2022-03-11

**Authors:** Clara Paz, Trinidad Abiuso, Lila Adana-Díaz, Alberto Rodríguez-Lorenzana, Tatiana Jaramillo-Vivanco, Esteban Ortiz-Prado, Ignacia Páez Monge, Guido Mascialino

**Affiliations:** ^1^ Escuela de Psicología y Educación, Universidad de Las Américas, Quito, Ecuador; ^2^ Universidad de Las Américas, Quito, Ecuador; ^3^ Grupo Bio-quimio Informática, Universidad de Las Américas, Quito, Ecuador; ^4^ One Health Research Group, Universidad de Las Américas, Quito, Ecuador; ^5^ National Department of Mental Health, Ministry of Public Health (Ecuador), Quito, Ecuador

**Keywords:** anxiety, pandemic, COVID-19, depression, Galapagos Islands

## Abstract

**Objectives:** to explore the emotional impact of the COVID-19 pandemic in the Galapagos Islands.

**Methods:** an online survey of 369 participants, conducted on October of 2020, was used to assess levels of depression, anxiety, and stress, as well as specific behavioral and emotional reactions to the pandemic.

**Results:** the prevalence of anxiety was 4% and depression 3.65%. Perceived stress level was higher, with 52% of the sample reporting moderate amounts. Women had higher levels of depression and perceived stress. Financial distress, interpersonal conflicts, feelings of isolation and fear of contagion of COVID-19 were all associated with higher levels of anxiety, depression, and stress.

**Conclusion:** prevalence of anxiety and depression is lower in the Galápagos Islands during the pandemic compared to other regions, while stress levels are more significant and may warrant intervention. Despite being low, anxiety and depression were associated with potentially problematic behaviors and emotional reactions.

## Introduction

The COVID-19 pandemic is by far the worst health crisis in recent times. According to the latest data, at least 190 million cases and 4.2 million deaths have been reported worldwide [1]. At a global level, the economic, social and health-related impact of the COVID-19 pandemic is extraordinary, and its consequences will continue to be felt well after infections are brought under control [2–4]. Measures taken in response to the pandemic halted or severely disrupted everyday activities, escalating unemployment, transforming education, and overall upending social interactions for indeterminate periods of time, leading some to question the cost-benefit ratio of those interventions [5].

The global impact of the pandemic on mental health has become one of the top priorities for health authorities. Anxiety, depression, and psychological distress have become more prevalent across many regions [6–8]. In a global sample of 1,612 people from Australia, China, Ecuador, Iran, Italy, Norway and the United States, Passavanti et al. [9] found symptoms of depression in 68.7% of the participants, among which 40.1% were in the moderate to severe range. Within the same sample, 44.7% presented some level of anxiety, with 39.5% corresponding to the moderate to severe range. In China, 52.8% of a general population sample indicated the presence of depressive symptomatology, while the presence of anxiety was observed in 46.7% of the participants [10]. These values contrast highly with respect to the 3.6% prevalence of depression and 5% of anxiety assessed before the outbreak [11].

Latin America is now considered by some authors to be one of the epicenters of the COVID-19 pandemic [12, 13]. Almost one third (32.53%) of all COVID-19 deaths to date occurred in the region [14], and within the most currently affected countries by COVID-19, Latin America occupies seven of the top ten spots in adjusted mortality [15]. Even though Latin America has been distinctly and severely affected by the pandemic, few studies have looked at its impact on the mental health of the population [16].

In Argentina, the levels of depression in the general population were measured during the first and second phases of the pandemic, revealing moderate/severe depression in 24.3% of the population in the first phase and an increase to 47.8% in the second phase [17] In Mexico, at the beginning of the health emergency, Cortés-Álvarez et al. [18] recorded a prevalence of moderate/severe depression of 15.7% and of moderate/severe anxiety of 22.6% in a sample of 1,105 participants from 32 Mexican states. Antiporta et al. [16] recorded in Perú levels of depression 5 times higher than those evaluated in 2018, with a rate of 34.9% compared to the 6.4% indicated before the pandemic.

Ecuador, within the Latin American region, was one of the countries most affected early on by the pandemic. High infection and death rates nearly collapsed a healthcare system already stretched thin for resources [19]. Several studies have explored the impact of the pandemic in this country, all of which were conducted in the first 7 months. Paz et al. [20], early in the pandemic, found 20.9% of people with suspected of confirmed COVID-19 had moderate to severe levels of depression, and 22.5% with moderate to severe anxiety symptoms. Caycho-Rodriguez et al. [21] found similar levels of clinically significant depression and anxiety (25.9% and 25.4% respectively) using the same instruments in an online survey of 790 participants in the community. Tusev et al. [22] found 25% reported clinically significant depression and 31.8% significant anxiety in an online survey using the 21 item depression, anxiety and stress scale (DASS-21). Passavanti et al. [23], in the above-mentioned seven multi-country comparison, found Ecuador had the highest levels of anxiety on the DASS-21, and high trauma scores in the IES-R were noted as well. Rodas et al. [24] found high mean level of depression using the Center for Epidemiological Studies Depression Scale (CES-D), but no prevalence was reported.

The Galápagos Islands is a region of Ecuador, located about 1,000 km west of the mainland, with a population of 25,244 inhabitants per the 2015 census [25]. The main economic activity in the islands is tourism, which contributes directly and indirectly to all the population [26]. Galapagos has some unique features within the Ecuadorian population. This coastal province has the highest standard of living in Ecuador and due to its geographic isolation, containment and mitigation strategies were implemented quicker than in the rest of the country [27]. During the first months of the pandemic, the government of Ecuador was facing the deadliest outbreak worldwide within some of the continental provinces such as Guayas, Santa Elena and Guayaquil, neglecting other provinces such as the Galapagos.

The first intervention undertaken within the island came with the collaboration of a local university which undertook the role of offering molecular diagnosis by RT-qPCR to the island [27]. During the first months of the pandemic, Galapagos only had the support from the local university and some of the local stakeholders that provided logistical support. After this, when the outbreak of the first wave was contained, the local government implemented additional testing capabilities, running free testing for every islander [28, 29]. The first cases in Galapagos were reported on March 24th by the ministry of health, totaling four infections (Table 1). Cases grew slowly and reached 227 by end of October with one confirmed death. In order to contain the spread, strict travel restrictions were implemented, local epidemiological surveillances was successfully put in place, and as soon as they were available vaccination campaigns were deployed. As a result Galapagos only reported 819 confirmed cases and 19 officially recorded deaths as of June 2021, while the country as a whole recorded 446,633 confirmed cases and 21,304 deaths [30].

**TABLE 1:** Galapagos cumulative cases and deaths due to COVID-19 from March 2020 to November 2020. Ministry of Public Health Situational Report, Galapagos, 2020.

Date	Cases	Deaths
3/31	5	0
4/30	68	1
5/31	76	1
6/30	88	1
7/31	103	1
8/31	109	1
9/30	198	1
10/31	227	1
11/30	628	2

Research about the Galapagos Islands appears to focus on the unique biology of the area, while its population is less often contemplated. To the best of the author’s knowledge, only two studies related to psychological functioning in the Galapagos Islands are present in the peer-reviewed literature: one looking at maternal stress and its impact on infant development [31] and another one surveying cognitive decline in the islands [32]. The importance of obtaining data on mental health on this locality is underscored by the islands limited resources and tourism-dependent economies that are particularly vulnerable to disruptive natural and social phenomena [33]. There is an urgent need to consider the well-being of the inhabitants in the face of crisis situations, such as the current pandemic, specifically by studying anxiety and depression, both of which can become elevated in the face of disasters [34].

To date, there are no indicators of the mental health of the inhabitants of the Galapagos islands, either before or during the pandemic. Thus, the present study aims to determine the levels of anxiety and depression in the context of the SARS-COV-2 pandemic in the general population of the Galapagos archipelago. This work can be seen as a continuation of the work by Paz et al. [20] and Paz et al. [35], which examined the emotional functioning of people in continental Ecuador but contained no information about inhabitants of the Galapagos Islands, because of a lack of reported COVID-19 cases in the islands at the time that study was conducted.

## Methods

### Design and Procedure

During the pandemic the Ecuadorian Ministry of Public Health (MoPH) conducted active surveillance of the emotional impact of the disease by developing an online self-reporting questionnaire to identify needs and provide treatment. The authors of this study were invited to collaborate in the development of this survey. The tool, described in the measures section, recorded sociodemographic variables, responses from the PHQ-9, GAD-7, PSS-10 and other behavioral and attitudinal variables. The link to the questionnaire was distributed at COVID-19 testing centers in the Galapagos Islands during the month of October 2020. At this time, participants were informed of the nature and purpose of the survey, including that participation was voluntary and responses would be kept anonymous. Once collected, and after serving its clinical purpose, the database was deidentified and made available for research purposes. The design of this study is thereby a retrospective analysis of this deidentified database.

### Participants

A total of 397 persons completed the online survey. Only participants who completed at least one measure of symptoms, and were at least 18 years-old, were included in the analysis, yielding a final sample size of 369.

### Ethical Considerations

This retrospective analysis of a deidentified database received approval from the Universidad de Las Américas Ethics Committee (#200301-001). The information from the online reports was collected and utilized by the MoPH to assess and address mental health needs during the pandemic. During that process, participants were informed about the purpose of data collection, ensured anonimity of the data, and explained that completing the online questionnaire was voluntary and not tied to services received. Once the data fulfilled its clinical use, it was anonymized and thus no personal information was available to the research team thereafter.

### Measures

#### Patient Health Questionnaire-9

This brief self-report questionnaire assesses the severity of depression symptoms in an adult population through nine items [36]. The instrument has demonstrated good test-retest reliability (*r* = 0.84) and good internal consistency (Cronbach’s α = 0.89). In the present study we used the Spanish version of the PHQ-9, which shows psychometric properties comparable to the original version [37]. The internal consistency of the scores in the present study was good (Cronbach’s *α* = 87). According to the scores given by the participants the symptoms can be classified in five levels: a score of between one and four points indicates the presence of minimal symptomology, between five and nine mild, 10 to 14 moderate, 15 to 19 moderately severe and 20 to 27 severe. A cut off score of 10 demonstrated 88% sensitivity and 88% specificity in the detection of Major Depressive Disorder [36].

#### Generalized Anxiety Disorder-7

This is a brief self-report questionnaire that assesses the presence and severity of symptoms related to Generalized Anxiety Disorder through seven items [38]. Each item is scored on a four-point Likert scale and the sum of scores is used to identify symptom severity. The severity of the symptoms is classified considering absence to scores lower than five, from five to nine, mild; from 10 to 14, moderate and greater or equal to 15, severe anxiety. A cut-off score of 10 showed 89% sensitivity and 82% specificity in the detection of Generalized Anxiety Disorder (Spitzer et al., 2006). The original version has presented good psychometric properties [38]. The Spanish version was used in this study, which also has good internal consistency [39]. The internal consistency of the scores in the present study was good (Cronbach’s *α* = 0.89).

#### Perceived Stress Scale-10

This is a 10-item self-reported questionnaire that assess the level of perceived stress in the last month [40]. Each item is scored in a five-point Likert scale from 0 to 4, excepting items 4, 5, 7 y 8 which are reverse scored. The scores given to the original version had presented good internal consistency (Cronbach’s *α* from 0.84 to 0.86). The Spanish version of the measure is used in the present study, which had presented good psychometric properties [41]. For the scores given in the present study the internal consistency was acceptable (Cronbach’s *α* = 0.67). The levels of perceived stress were classified as follows, scores from 0 to 13 as mild stress, from 14 to 26 as moderated stress and from 21 to 40 as high perceived stress.

#### Behaviors and Personal Reactions to the COVID-19 Pandemic

These six items, derived through expert consensus by five psychologists, were developed to capture specific behaviors and reactions of the participants in relation to COVID-19 pandemic. Answers to the items are dichotomous. The questions covered information about the presence or absence of financial distress, increased alcohol consumption, increased interpersonal conflicts, feelings of isolation, fear of contagion of COVID-19, and avoidance of leaving home in response to the pandemic.

### Data Analysis

Required sample size was calculated using Cochran’s [42] formula and estimated to be 379. Descriptive statistics were used to characterize sociodemographic and clinical variables. Differences in clinical scales total scores related to demographic groups and endorsement of behavioral/emotional problems were analyzed using *t*-tests for variables with two levels analyses of variance (ANOVA) for variables with more than two levels. Mann-Whitney U test was utilized for non-parametric analyses. All tests were two-tailed, and significance level was set up at *p* < 0.05. Hedges g and Eta^2^ were used as estimates of effect size for t-tests and ANOVAs respectively. In order to estimate effect size for Mann-Whitney U test, the normal approximation method of z to r described by Pallant [43] was utilized. All the analyses were conducted using R [44].

## Results

In total, 369 persons participated in the study that answered at least one of the outcome measures (PHQ-9, GAD-7 or PSS-10), 72.6% of which (*n* = 268) were males. Age ranged from 18 to 66 years with a mean of 35 (*SD* = 9.60); most of the participants were within 18–35 (58.3%, *n* = 215), 34.7% (*n* = 128) within 26–53 years and 7% (*n* = 26) were older than 53 years. Of the total sample, 356 provide information to calculate a score for the PHQ-9, resulting in a mean total score of 2.18 (SD = 3.47). A total of 361 participants had enough data to calculate a score of the GAD-7, resulting in a mean score of 2.57 (SD = 3.42). Finally, 365 participants provided enough data to calculate a PSS-10 total score, resulting in a mean score of 13.6 (SD = 5.60). The prevalence by severity of each of the assessed symptoms is presented in Table 2. Prevalence of clinically significant anxiety (GAD-7 ≥ 10) was 4%, and prevalence for depression was 3.65% (PHQ-9 ≥ 10). Table 3 presents t-test, ANOVA, and Mann-Whitney U test analyses contrasting sociodemographic variables and scores on the PHQ-9, GAD-7 and PSS-10, as well as their relationship to the six aforementioned questions related to various behavioral and emotional reactions to the pandemic. There were significant gender differences for symptoms of depression and perceived stress, with women scoring higher. Also, higher scores on the PHQ-9, GAD-7 and PSS-10 were associated with the presence of financial distress, interpersonal conflicts, feelings of isolation and fear of contagion of COVID-19. Finally, those who reported avoiding leaving home presented with lower levels of stress.

**TABLE 2:** Prevalence by severity of symptoms. Psychological distress in Galapagos due to COVID-19, Galapagos, 2020.

	None or minimal % (*n*)	Mild % (*n*)	Moderate % (*n*)	Moderately severe % (*n*)	Severe % (*n*)
Depression	83.5 (297)	12.9 (46)	2.0 (7)	0.8 (3)	0.8 (3)
Anxiety	76.7 (277)	19.11 (69)	2.4 (9)		1.6 (6)
Perceived Stress	46.8 (171)		52.6 (192)		0.5 (2)

**TABLE 3:** Means, standard deviations, and test results for each demographic and behavioral reaction to COVID-19 pandemic by score of symptoms for the PHQ-9, GAD-7 and PSS. Psychological distress in Galapagos due to COVID-19, Galapagos, 2020.

Variable	*n* (%)	PHQ-9				GAD-7				PSS-10			
		M(SD)	t/F/U	*p*	Hedges g/Eta^2^/r	M(SD)	t/F/U	*p*	Hedges g/Eta^2^/r	M(SD)	t/F/U	*p*	Hedges g/Eta^2^/r
Sex			2.97	0.003*	0.364		1.89	0.06	0.24		2.76	0.006*	0.312
Women	101 (27.4)	3.09 (3.60)				3.16 (3.82)				14.8 (5.22)			
Men	268 (72.6)	1.84 (3.36)				2.35 (3.23)				13.1 (5.67)			
Age			1.41	0.25	0.008		0.06	0.94	0.0003		1.02	0.36	0.006
18–35	215 (58.3)	2.44 (3.51)				2.56 (3.09)				13.9 (5.60)			
36–53	128 (34.7)	1.88 (3.42)				2.55 (3.41)				13.0 (5.33)			
>53	26 (7.0)	1.6 (3.27)				2.8 (5.66)				13.4 (6.76)			
Financial distress			−3.85	<0.001**	−0.38		−3.78	<0.001**	−0.392		−5.07	<0.001**	−0.547
Absence	145 (39.3)	1.40 (2.39)				1.79 (2.95)				11.8 (5.52)			
Presence	219 (59.3)	2.71 (3.69)				3.11 (3.63)				14.8 (5.37)			
Missing	5 (1.4)	1.25 (1.5)				1.67 (2.21)				15.0 (4.12)			
Increased alcohol use			1923	0.563	0.032		2,910	0.092	0.091		3,076	0.035*	0.195
Absence	347 (94.0)	2.09 (3.24)				2.49 (3.28)				13.5 (5.59)			
Presence	14 (3.8)	4.69 (7.58)				4.86 (5.95)				16.4 (5.87)			
Missing	8 (2.2)	2.38 (2.67)				2.10 (2.29)				14.3 (4.50)			
Interpersonal conflicts			4,421	<0.001**	0.21		5,078	<0.001**	0.213		4,981	<0.001**	0.17
Absence	338 (91.6)	1.98 (3.19)				2.31 (3.05)				13.3 (5.42)			
Presence	22 (6.0)	5.95 (5.75)				6.89 (5.64)				18.6 (6.63)			
Missing	9 (2.4)	1.22 (1.48)				1.74 (2.33)				13.6 (3.70)			
Feelings of isolation			−3.3	0.001*	0.447		−4.34	<0.001**	−0.543		−6.15	<0.001**	−0.447
Absence	238 (64.5)	1.70 (2.67)				1.98 (2.92)				12.4 (5.57)			
Presence	120 (32.5)	3.24 (4.62)				3.80 (4.04)				16.0 (4.99)			
Missing	11 (3.0)	1.5 (1.53)				1.9 (2.43)				13.1 (4.18)			
Fear of contagion			−2.84	0.005*	−0.298		−2.16	0.03*	−0.241		−4.23	<0.001**	−0.459
Absence	146 (39.6)	1.58 (2.98)				2.08 (3.71)				12.1 (5.59)			
Presence	210 (56.9)	2.62 (3.77)				2.91 (3.18)				14.6 (5.42)			
Missing	13 (3.5)	1.78 (2.20)				2.56 (3.35)				14.2 (4.99)			
Avoid leaving home			0.91	0.36	0.098		0.28	0.83	0.023		2.15	0.03*	0.225
Absence	172 (46.6)	2.33 (3.58)				2.58 (3.08)				14.2 (5.15)			
Presence	192 (52.0)	2.00 (3.33)				2.51 (3.71)				13.0 (5.96)			
Missing	5 (1.4)	4.4 (4.39)				4.6 (2.97)				14.5 (3.51)			

Note: PHQ-9, patient health questionnaire; GAD-7 = generalized anxiety disorder = 7, PSS-10, Perceived Stress Scale-10.

* = *p* < 0.05, ** = *p* < 0.001

## Discussion

This is the first study reporting on the mental health status of the general population in the Galápagos Islands during the COVID-19 pandemic. While approximately 17%–23% of the sample showed at least mild symptoms of depression and/or anxiety, only a small proportion of the participants endorsed enough symptoms to be considered clinically problematic. Just 4% of the participants presented moderate to severe symptoms of anxiety, and 3.65% reported moderate to severe symptoms of depression. However, more than half the sample reported moderate levels of perceived stress in response to the pandemic.

The prevalence of anxiety and depression found in this study is considerably lower than what has been published to date. Recent meta-analyses present a prevalence of anxiety between 16.6% and 32.6%, and depression between 27.6% and 37.7%, depending on the study [45–47]. Furthermore, prior studies of emotional functioning during the pandemic in Ecuador also showed higher prevalence of psychological problems; Paz et al. [20], utilizing a methodology similar to the current study but including information only about continental Ecuador, found a prevalence of 20.9% for depression and 22.5% for anxiety early in the pandemic, on March of 2020). Passavanti et al. [9] found a mean score of 8.75 on the PHQ-9 for Ecuadorian participants in a multi-country study, compared to a PHQ-9 mean score of 2.18 on the current one. Levels of perceived stress in this study appear to be more indicative of psychological distress, with 52% reporting moderate levels. However, results are also lower than those reported by Passavanti et al. [9] with an Ecuadorian sample, who found a higher mean level of perceived stress on the PSS-10 than the current study (19.25 vs. 13.60). In addition, the percentage of persons presenting low levels of stress was higher in our study (46.3%) that in Passavanti et al. [9]. Rodas et al. [24] administered the CES-D to a sample of 663 participants in Ecuador during the early stages of the pandemic (March to June) and, although prevalence scores for depression were not reported, the sample average was 19.6 (SD = 11.05), which is above the suggested cut-off score of 18 for the detection of depression. Furthermore, Tusev et al. [22] and Caycho-Rodriguez et al. [21] both found higher levels of significant depression (25% and 25.9% respectively) and anxiety (31.8% and 25.4% respectively) in large online samples.

Several factors may account for the stark differences in symptomatology between other regions and the Galapagos Islands. The existing studies focused heavily on the early months of the pandemic: four of five took samples between March and June exclusively. These were considered the most difficult months in the country with regards to cases and deaths at the national level [48]. The current study, however, looked at data obtained in October. In addition, cases and deaths in Galapagos were lower than the peaks experienced in large rural areas like Guayaquil and Quito [48]. As of the end of October of 2020, the Galapagos Islands reported 227 cases and one death. It is possible the psychological impact is thus lower because of these values. Higher stress levels identified, however, may be a response to the economic pressures the islanders were suffering due to the restrictions, which is illustrated by the statistically significant relationship found between self-reported economic problems and levels of stress. Regardless of lower levels of symptom report of anxiety and depression found in this study, perceived stress might be considered for future interventions in the Galápagos Islands. More than a half of the participants reported moderate levels of perceived stress. It is also relevant to note that PHQ-9 and GAD-7 are self-reports measures for the past 2 weeks, while the PSS-10 asks to report feelings and thoughts in the last month, probably capturing a longer lasting mental health state.

Analysis of sociodemographic effects on symptomatology revealed a gender effect on levels of depression, as did other studies during the pandemic in which women presented with higher levels of depression than men [6]. This is not surprising given that, even prior to the pandemic, gender differences have been reported around the world [49] Women also presented with higher levels of perceived stress in this study, consistent with results from a 48-country study of perceived stress during the pandemic [50]. Hidalgo-Andrade et al. [51] also found higher levels of perceived stress in Ecuadorian female teachers during the pandemic. Gender differences in stress levels were thought to be related to unequal domestic task distribution in that study, as participants who reported being responsible for taking care of children and/or older adults presented with higher levels of distress and perceived stress. Other variables previously found to be associated with psychological functioning during the pandemic include age, income, and educational level. Present results did not show an association between age and psychological functioning, while other sociodemographic variables were not included in the analysis. Lastly, it is unclear why those who endorsed avoiding leaving home had lower levels of stress, but it may reflect reduced fear of contagion and feelings of safety at home.

In this study we also tried to identify problematic behaviors and emotional states that might relate to levels of depression, anxiety and stress. The presence of financial distress, interpersonal conflicts, feelings of isolation and fear of contagion of COVID-19 were associated with higher scores for the PHQ-9, GAD-7, and PSS-10. These behaviors and reactions should be considered when preparing interventions for this population and thus constitute possible target areas. Stress inoculation, interpersonal conflict resolution, and emotional support interventions may be appropriate to address these issues. Of note, while symptom report was lower than anticipated in this study, its relationship to problematic behavioral and emotional phenomena underscores the need to address psychological problems during the pandemic.

This study presents with some limitations. This is a cross-sectional study, thus capturing the mental state of the population at the moment the survey was completed. As the personal, social and health status of the inhabitants of the Galapagos Islands change in reaction to shifting conditions during the pandemic, follow up surveys might help to understand fluctuations in psychological symptoms. As with all studies using online data collection, certain limitations may apply such as the effect of limited computer literacy and differences in user engagement brought about by the medium [52].

### Conclusion

This is the first study exploring levels of anxiety, depression, and stress during the pandemic in the Galápagos Islands. Anxiety and depression were markedly lower than in mainland Ecuador per comparison with recent studies, although the timing of data collection may be in part responsible for these differences. It is also likely less significant infection and mortality in the Galapagos Islands contributed to lower levels of psychopathology, which are possibly close to baseline. However, perceived stress levels were higher and this may in part be related to the changes in everyday life related to pandemic restrictions, including economic distress. In addition, certain problematic behaviors and emotional reactions are associated with higher symptom load overall, most notably financial distress, increased personal conflicts, feelings of isolation and fear of contagion. This information can help shape interventions to address psychological distress in the midst of a pandemic. Further studies are needed to understand the mental health status of this population and the possible fluctuations associated with the pandemic.
